# Intramolecular bridging strategies to suppress two-phonon Raman spin relaxation in dysprosocenium single-molecule magnets†

**DOI:** 10.1039/d4cp01716a

**Published:** 2024-06-10

**Authors:** Jakob K. Staab, Md. Kholilur Rahman, Nicholas F. Chilton

**Affiliations:** a Department of Chemistry, The University of Manchester Manchester M13 9PL UK; b Research School of Chemistry, The Australian National University Canberra 2601 ACT Australia nicholas.chilton@anu.edu.au

## Abstract

Dy(iii) bis-cyclopentadienyl (Cp) sandwich compounds exhibit extremely strong single-ion magnetic anisotropy which imbues them with magnetic memory effects such as magnetic hysteresis, and has put them at the forefront of high-performance single-molecule magnets (SMMs). Owing to the great success of design principles focused on maximising the anisotropy barrier, ever higher *U*_eff_ values have been reported leading to significant slow down of single-phonon Orbach spin relaxation. However, anisotropy-based SMM design has largely ignored two-phonon Raman spin relaxation, which is still limiting the temperatures at which a memory effect can be observed. In this work, we study the suppression of Raman relaxation through covalent bridging of the Cp ligands by alkyl chains, testing the hypothesis that increasing the rigidity of the ligand framework results in a blue shift of low frequency vibrations in the first coordination sphere of the Dy(iii) ion. This reshaping of the vibrational low-energy density of states (DOS) results in lower occupation of pseudo-acoustic phonons available to drive Raman relaxation at low temperatures. We simulate Orbach and Raman spin relaxation in a series of zero-, mono-, di- and tri-bridged [Dy(Cp^ttt^)_2_]^+^ analogues fully *ab initio*, using a quantum mechanics (QM)/molecular mechanics (MM) condensed phase embedding protocol in a periodic solvent matrix as a generic and experimentally testable environment model that can include (pseudo-)acoustic phononic degrees of freedom. We show that this approach can simulate magnetic relaxation dynamics in the condensed phase for the existing non-bridged [Dy(Cp^ttt^)_2_]^+^ compound with quantitative experimental accuracy. Subsequently, we find a significant slowing down of Raman relaxation can be achieved for the singly-bridged SMM, while the introduction of further bridges leads to faster relaxation. A key result being that we find the two-phonon Raman rates correlate with the purity of the first-excited Kramers doublet in terms of its *m*_J_ = ±13/2 content. Even though the bridging design principle is successful at progressively reshaping the low-energy DOS, the introduction of linker atoms in the equatorial plane successively degrades magnetic anisotropy, suggesting the importance of refined design of the linker chemistry. The accuracy of our results emphasises the value of a generic periodic solvent embedding model, such that it permits the modelling of molecular spin dynamics in the condensed phase without knowledge of a crystal structure. This allows the study of hypothetical molecules or aggregates under real-world conditions, which we expect to have utility beyond the field of molecular magnetism.

## Abstract 2

Generic frozen solution embedding enables the ab initio prediction of condensed-phase spin-dynamics under realistic conditions for molecules without crystal structures, giving insight into the impact of intramolecular bridging on magnetic relaxation.

## Graphical abstract

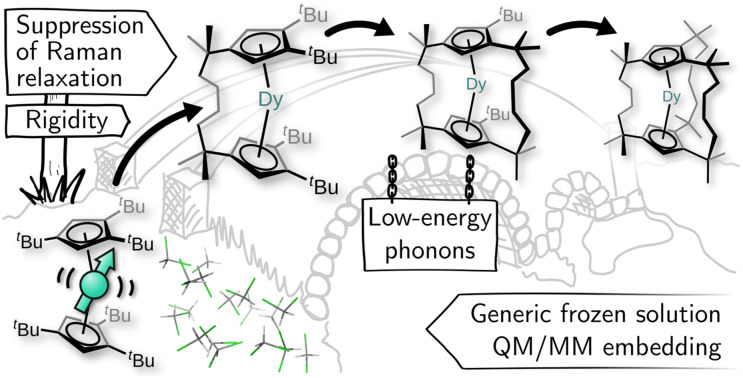

## 1 Introduction

The family of cationic Dy(iii) bis-cyclopentadienyl (Cp) sandwich single-molecule magnets (SMMs) has attracted much attention in recent years due to their relatively long magnetic memory time, making them possible candidates for application in high-density data storage devices.^1–7^ The magnetic memory timescale in SMMs is generally limited by loss of magnetisation *via* spin–phonon coupling, which drives the exchange of energy between the thermally populated phonons in the environment and the magnetic spin states. The exceptionally slow magnetic relaxation in [Dy(Cp^R^)_2_]^+^-type SMMs occurs due to their strong axial ligand field with no equatorial interactions, and a rigid first coordination sphere.^1,8,9^ This ligand environment generates a large energy barrier towards Orbach magnetic reversal (which implicates numerous single-phonon interactions) and suppresses the two-phonon Raman-like magnetic reversal mechanism, which had previously limited memory times.^10^ Much computational and experimental effort has been put towards enhancing magnetic memory timescales by enlarging the Orbach energy barrier by tweaking the Cp substituents in a trade-off between more linear Cp^R^–Dy–Cp^R^ angles and shorter Dy–Cp^R^ distances.^2,4–7,11,12^ However, anisotropy-focussed engineering strategies of substituted [Dy(Cp^R^)_2_]^+^ complexes, even when considering strong exchange coupling,^13^ seem to have approached a limit with record barrier heights obtained of 1541(11) cm^−1^ for [(Cp*)Dy(Cp^iPr5^)][B(C_6_F_5_)_4_]^4^ and 1631(25) cm^−1^ for Cp^iPr5^Dy_2_I_3_.^13^ Furthermore, such anisotropy-focussed design considerations have largely ignored the low-temperature barrier-less two-phonon Raman mechanism which is driven by low-energy (pseudo-)acoustic phonons, and limits the magnetic memory lifetime at technologically and economically viable operating temperatures.

**Fig. 1:**
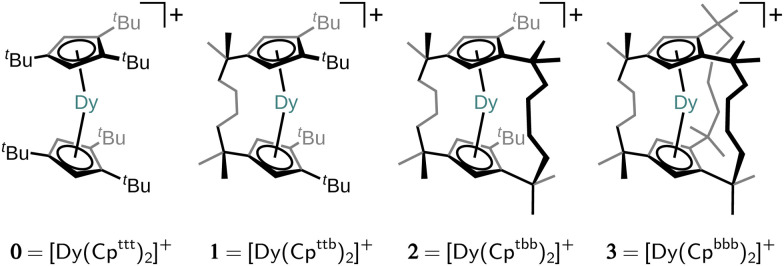
Chemical structures of the parent molecule [Dy(Cp^ttt^)_2_]^+^ (0) and its ***n***-bridged analogues obtained by linking neighbouring *tert*-butyl groups [(Cp^Rt^)_2_]^2−^ = [Cp^R^-CMe_3_ Me_3_C-Cp^R^]^2−^ to yield the bridging motif [(Cp^Rb^)_2_]^2−^ = [Cp^R^-CMe_2_(CH_2_)_4_CMe_2_-Cp^R^]^2−^.

In this work, we investigate a design strategy based on covalent alkyl bridging between the Cp^R^ ligands. The aim of this strategy is to preserve the axiality of the Cp^R^ ligands whilst removing low-energy degrees of freedom in the ligand–Dy(iii) system by systematically suppressing pseudo-acoustic vibrations that may occur between the components of the complex. A very similar strategy has been recently investigated by Kotrle *et al.*^14^ who estimated effective anisotropy barriers *U*_eff_ in a series of [Dy(Cp^R^)_2_]^+^ compounds with varying numbers of Me substituents and –CH_2_CH_2_– linkers. Their study was based on estimation of *U*_eff_ from magnetic dipole matrix elements and intramolecular vibrations in the gas phase. Here, we examine a different bridging strategy, *viz.* a series of singly-, doubly- and triply-bridged SMMs, [Dy(Cp^ttb^)_2_]^+^ (1; [Cp^ttb^]^−^ = C_5_H_2_-1,2-^*t*^Bu_2_-4-[CMe_2_CH_2_CH_2_]^−^), [Dy(Cp^tbb^)_2_]^+^ (2; [Cp^tbb^]^−^ = C_5_H_2_1-^*t*^Bu-2,4-[CMe_2_CH_2_CH_2_]_−2_) and [Dy(Cp^bbb^)_2_]^+^ (3; [Cp^bbb^]^−^ = C_5_H_2_-1,2,4-[CMe_2_CH_2_CH_2_]_−3_), derived from the parent molecule [Dy(Cp^ttt^)_2_]^+^ (0; [Cp^ttt^]^−^ = C_5_H_2_-1,2,4-^*t*^Bu_3_) shown in Fig. 1, and, crucially, go one step further by predicting the full one- and two-phonon spin dynamics with state-of-the-art computational methodologies.^15,16^ Thus, we are able to assess how linking of the two Cp^R^ rings in [Dy(Cp^R^)_2_]^+^-type SMMs impacts the Raman relaxation regime. As the two-phonon Raman mechanism relies on low-energy acoustic phonons which only exist in the solid state, we must include a chemically-reasonable environment in which to embed the hypothetical compounds. We propose a flexible computational approach which embeds a single SMM into a periodic frozen solution environment, circumventing the explicit presence of the counter anion and the knowledge of the crystal structure. To this end, we introduce a quantum mechanics (QM)/molecular mechanics (MM) model (for SMM/solvent components) capitalising on recent theoretical and methodological advances by us and others for the computationally efficient and accurate analytic evaluation of spin–phonon couplings in the condensed phase.^15–17^ Critically, our methodology mimics experimentally-testable conditions so that our predictions herein may be explored in the lab by synthetic colleagues. We find that the bridging strategy successfully enhances the rigidity of the ligand–Dy(iii) system, while also constraining it to a more linear Cp^R^–Dy–Cp^R^ angle, resulting in significantly slower Raman and Orbach relaxation in 1, characterised by a drop in the Raman power-law prefactor and enhancement of the Orbach barrier height, respectively. However, the addition of further linkers in 2 and 3 leads to increased Dy–Cp^R^ distance and progressively detrimental equatorial interactions with the metal, reducing axial magnetic anisotropy and thus resulting in faster spin dynamics. Thus, our results suggest there is merit in the overall strategy, but call for a more refined linker design.

## 2 Computational details

The relaxation dynamics of compounds 0–3 in frozen dichloromethane (DCM) solution were computed following a four-step protocol: (i) structure generation, (ii) geometry optimisation and harmonic analysis of nuclear vibrations at the density-functional theory (DFT)/MM level of theory, (iii) computation of complete active space self-consistent field (CASSCF) electronic states and spin–phonon couplings, and (iv) evaluation of semi-classical spin dynamics. While steps (ii) through (iv) are well-established in the literature,^1,12,15–21^ this work is the first of its kind involving the embedding into an explicit periodic frozen solution environment for the inclusion of a generic pseudo-acoustic phonon bath into a spin-dynamics calculation.

To generate the initial molecular structure of compound 1, we insert a covalent ethyl linker between the methyl groups of opposing *tert*-butyl substituents (based on a minimum distance criterion), starting from the gas-phase DFT optimised crystal structure^1^ of the non-bridged parent molecule 0. The structure of 1 was then optimised with DFT (see details below) in the gas phase. We then build compound 2 by adding another ethyl linker to 1 following the same methodology and optimising its geometry, and subsequently building 3 by adding another ethyl linker to 2 and optimising its geometry. We then embed each pre-optimised structure at the centre of a cubic solvent box of dimensions 20 Å × 20 Å × 20 Å filled with 100 DCM molecules (removing any that clash), and perform a two-step molecular dynamics (MD) simulation using the isobaric isothermal ensemble at 1 bar using a barostat coupling of *τ*_p_ = 1.0 ps and a 1 fs time step. Here, the coordinates of the SMM are fixed to their initial gas-phase positions, which allows us to equilibrate the solvent packing and box dimensions. This is first run for 50 000 steps at a finite target temperature of *T* = 10 K under relatively strong thermostat coupling with a time-constant of *τ*_T_ = 0.1 ps and subsequently for 100 000 steps under weak coupling *τ*_T_ = 2.0 ps for complete freezing of the system to a target temperature of *T* = 0 K, yielding an approximate steepest decent optimised configuration of the solvent molecules around the gas-phase geometry of the SMM at absolute zero and ambient pressure. Finally, the full system system is optimised until self-consistency at fixed box size with alternating complete solvent relaxations followed by 10 steps of SMM optimisation, employing geometrical constraints on the SMM and the solvent, respectively, speeding up convergence. The optimisation is terminated upon reaching a maximum gradient of 0.001 kcal mol^−1^ Å^−1^ in the solvent and RMS (maximum) thresholds in force and displacement corresponding to 0.00045 a.u. (0.0003 a.u.) and 0.0018 a.u. (0.0012 a.u.) in the SMM optimisation, respectively. At the stationary point, harmonic frequencies and normal modes are computed from the full Hessian matrix. As the frozen solution-state system is periodic in the MM potential, only the three rigid translations of the whole box leave its energy invariant, *i.e.* as the *Γ*-point acoustic modes they do not represent vibrational degrees of freedom (DOFs) and thus have vanishing vibrational frequencies. However, the three rigid rotations of the box are associated to an energy change due to nuclei in neighbouring periodic images moving in opposite direction at the box boundaries. The three rigid translational DOFs are removed by first transforming the mass-weighted Hessian matrix into the orthogonal complement basis to the three translational mass-weighted displacement vectors, and then diagonalising the transformed Hessian yielding 3*N*_atoms_ − 3 vibrational frequencies and normal modes which are guaranteed to lack any rigid translational component.^22^

Throughout these steps, a DFT/MM hybrid potential is set up using the ONIOM^23^ scheme for describing all interaction within the QM region represented by the SMM with DFT and all interactions within the classical region represented by the solvent with a classical force field, while the SMM is embedded mechanically *via* its classical force field interactions with the solvent. All gas-phase and DFT/MM single-point calculations, geometry optimisations and Hessian calculations are carried out using Gaussian version 9 revision D.01^24,25^ along with a modified version of the Garleek^26^ interface software to enable the external evaluation of the periodic MM potential energy, forces and Hessian using the Tinker MM-suite. The PBE functional^27^ supplemented by Grimme's D3 dispersion correction^28^ is used for all DFT calculations, employing Dunning's cc-pVDZ basis set^29^ on all atoms but the first coordination shell and the central Dy(iii) ion, which are instead equipped with the higher quality cc-pVTZ analogue as well as the 4f-in-core ECP55MWB basis set^30,31^ to avoid a multi-configurational ground state. Generic OPLS-AA^32^ force field parameters are used to model bonded and van der Waals (vdW) MM interactions (see ESI† for details). Revised atomic charges of DCM^33,34^ and explicitly computed gas-phase CHELPG^35^ charges on the SMM are employed within the particle mesh ewald (PME) method to describe the electrostatics; this method allows us to treat a cationic molecular system where the non-zero periodic charge is counterbalanced by a uniform negative charge throughout the cell.

The spin states and spin–phonon coupling of the electrostatically embedded dysprosocenium SMMs is computed at the CASSCF level including scalar relativistic effects using the second-order Douglas–Kroll Hamiltonian and spin–orbit coupling (SOC) through the the atomic mean-field integral (AMFI) approximation implemented in in the restricted active space state interaction (RASSI)^36,37^ approach implemented in OpenMolcas version 23.02.^38^ The dysprosium atom is equipped with the ANO-RCC-VTZP basis set, the ring carbons with the ANO-RCC-VDZP basis set and the remaining atoms with the ANO-RCC-VDZ basis set.^39^ The resolution of the identity (RI) approximation with the acCD auxiliary basis is employed to handle the two-electron integrals.^40^ The active space consists of 9 electrons in 7 orbitals, spanned by 4f atomic orbitals, and we perform a state-average CASSCF calculation for the 18 lowest lying sextet roots which span the ^6^H and ^6^F atomic terms. Exclusion of the ^6^P states, as well as all quartets and doublets, is made based on the significant energetic separation from the low-lying ^6^H and ^6^F terms. The solvent environment is included in our CASSCF calculations through their atomic MM charges (see ESI,† Table S2) complemented with a perfect conductor (*ε* → ∞) Kirkwood reaction field^41^ to correct the long-range electrostatics by compensating any residual non-zero multipole moments of the system (up to hexadecapole, including the positive net charge) representing a finite size slab of the infinitely periodic condensed phase system.^16^

Spin dynamics calculations were carried out using the TAU^42^ software, implementing a semi-classical approach used and described in previous work.^15^ Transition rates between different states are obtained by integrating the spin-one-phonon and spin-two-phonon rate expressions over the phonon density of states, weighted by Bose–Einstein occupation factors. Transitions among all states constituting the ^6^H_15/2_ multiplet driven by the whole vibrational spectrum are considered in case of the single-phonon Orbach rates, while only transitions within the ground Kramers doublet (KD) as driven by pairs of phonons with energy *ν̃* < 300 cm^−1^ are considered for the two-phonon Raman rates (though all states interact *via* the two-phonon expressions); the restriction of phonon energy avoids divergences arising from resonances with the energy gap to the first excited KD and does not impact the calculated rates at low temperatures where the Bose–Einstein occupation factors are vanishing for higher energy phonons. The spin–phonon matrix elements are evaluated in the equilibrium crystal field eigenbasis, using crystal field parameter (CFP) derivatives in normal mode coordinates, evaluated using our python packages spin–phonon-suite^43^ version 1.5.0 and angmom-suite^44^ version 1.17.2. The vibrational density of states (DOS) is constructed from harmonic frequencies broadened by antilorentzian lineshapes of a constant linewidth parameter FWHM = 10 cm^−1^.^16^

## 3 Results and discussion

### 3.1 Structure and rigidity

**Fig. 2:**
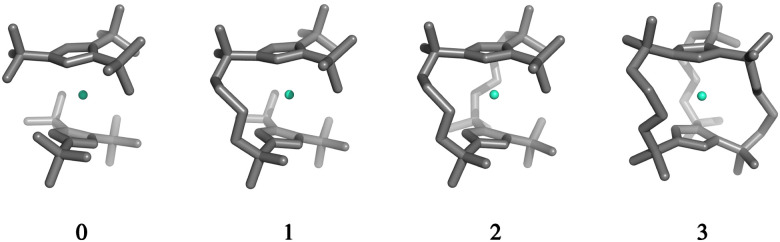
Gas-phase geometries of the non-bridged parent dysprosocenium 0 and its ***n***-bridged analogues 1–3.

The structures 1–3 have been generated by subsequently replacing spatially close *tert*-butyl hydrogens on opposite Cp^ttt^ ligands by covalently linked, saturated carbon chains (Fig. 2), which we propose could in principle be prepared *via* sequential ring-closing metathesis reactions between two identical alkene-tethered Cp^R^ ligands as shown in Fig. S1 of the ESI.† The resulting bridges lead to structural changes of the coordination geometry through two competing mechanisms: attraction of the Cp^R^ ligands due to possible strain of the alkyl bridges and repulsion through the steric bulk introduced in the equatorial plane of the complex. Since the alkyl bridges permit enough flexibility to avoid clamping of the Dy(iii) ion by the Cp^R^ ligands, the steric effects of the bulky alkyl linkers dominate which is observed in the progressive elongation of the average Dy–Cp^R^ centroid distance from 2.340 Å in the parent complex 0 to 2.357, 2.364 and 2.387 Å in complexes 1–3, respectively. At the same time, the Cp^R^–Dy–Cp^R^ angle increases from 151.3° in 0 to 156.1°, 161.7°, 162.7° (1–3). This is in contrast to the observations of Kotrle *et al.,*^14^ who observed a reduction in Dy–Cp^R^ centroid distances with an increasing number of bridges, along with highly strained and bent structures. This occurred in that case due to the use of a much shorter aliphatic linker, giving a total of four carbon atoms between the Cp^R^ rings, compared to bridges consisting of six carbon atoms used here.

While an increase in the Cp^R^–Dy–Cp^R^ angle is imperative to the discussion of Orbach relaxation, the main objective of this work is to investigate if Raman relaxation can be suppressed by enhancing the mechanical rigidity of the complex by ligand bridging. Unlike structural metrics, rigidity is not a static property of a given molecular structure, but instead is a measure of atomic forces resisting nuclear distortion away from the equilibrium geometry. Within the harmonic approximation of the potential energy surface (PES) on which the nuclei move, normal mode coordinates present a natural basis for investigating the rigidity of structural distortions.^45^ In this context, rigidity induced by ligand bridging can be observed as a blue-shift of the low energy vibrations between the ligands and the Dy(iii) ion, which has been identified as a contributing factor for slow Raman relaxation in SMMs featuring a rigid coordination sphere.^9,19^

**Fig. 3:**
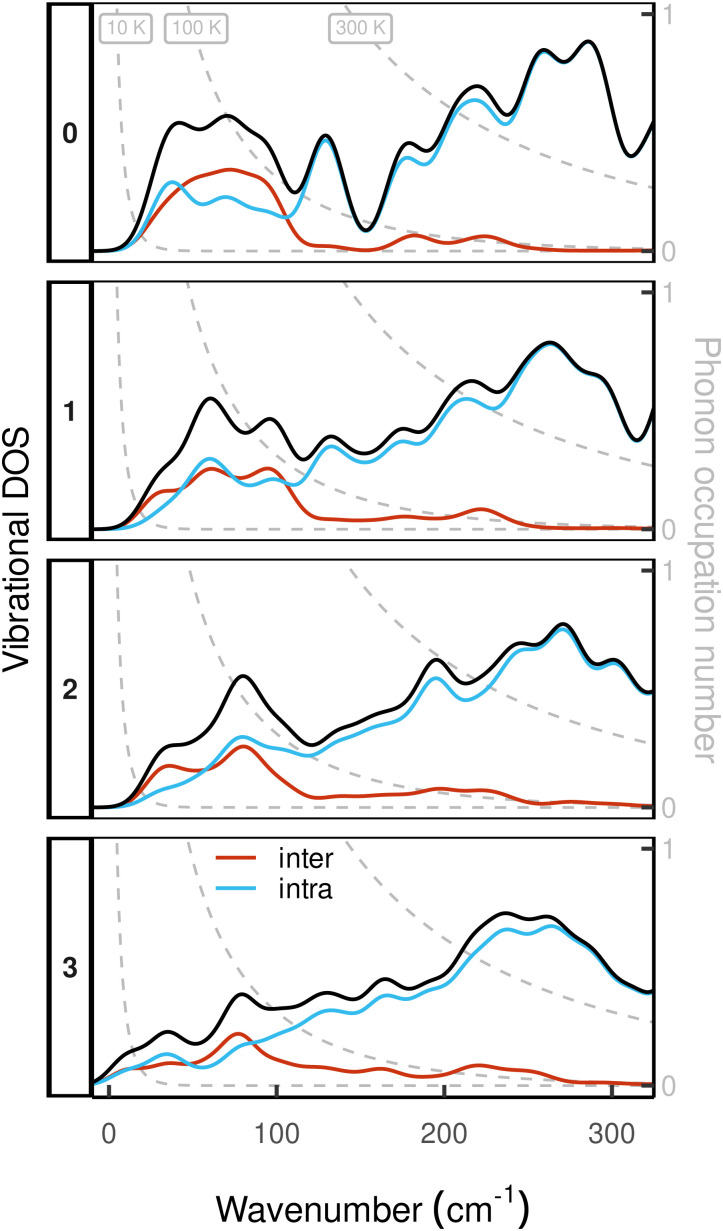
Decomposition of the low energy (*ν̃* = 350 cm^−1^) gas-phase vibrational DOS of compounds 0–3 into inter- and intra-fragment contributions. The DOS is computed as the sum of all harmonic frequencies dressed with a Gaussian of bandwidth 10 cm^−1^. The dashed, gray lines show the frequency dependent occupation of phonons at 10 K, 100 K and 300 K on the right axis underlining the relevance of low-energy vibrations for spin–phonon coupling driven electronic excitations at low temperatures.

To assess the mechanical rigidity of the series 0–3, the low-energy vibrational DOS of the gas-phase structures shown in Fig. 3 is analysed. Two effects can be observed; a general blue-shift of the low energy DOS in the region of *ν̃* ≤ 150 cm^−1^ and the build-up of a shoulder at the very low energy end of the vibrational DOS with increasing number of bridges. To interrogate the origins of these features, we use vibrational projection to decompose the total DOS into contributions originating from inter-fragment rigid body motion of the complex ligand and metal fragments, *i.e.*, the Dy(iii) ion and the two Cp^R^ ligands, as well as intra-fragment vibrational contributions in the ligands. To this end, orthonormalised mass-weighted displacements of the nine inter-fragment coordinates 
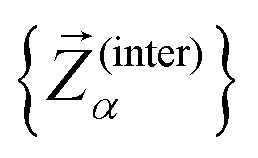
 were constructed, which consist of three rotational and three translational DOFs per Cp^R^ ligand, three Dy(iii) translations, and less the rigid body motions of the whole SMM, *i.e.* 1 ≤ *α* ≤ 2 × (3 + 3) + 3 − 6. Similarly, we construct the intra-fragment coordinates 
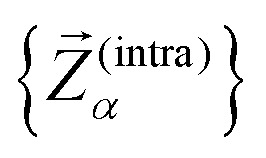
, which consist of three translations per SMM atom minus the rigid body motion and the inter-molecular DOFs, *i.e.* 1 ≤ *α* ≤ 3 × *N*^(ligand)^_atoms_ − (6 + 9). These sets are then projected against the mass-weighted normal mode coordinates 
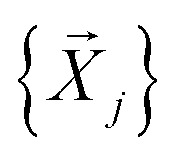
, again discarding rigid body translation and rotation (*i.e.* 1 ≤ *j* ≤ 3 × *N*_atoms_ − 6). In other words, a given normal mode displacement *X⃑*_*j*_ is expanded in terms of inter- and intra-fragment displacement vectors which span the same 3 × *N*_atoms_ − 6 dimensional space:1
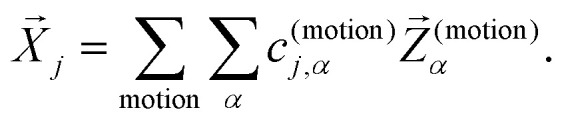


The expansion coefficients *c*^(motion)^_*j*,*α*_ can be derived by taking the dot product with a given *Z⃑*^(motion)^_*α*_ from the right:

2 *X⃑*_*j*_·*Z⃑*^(motion)^_*α*_ = *c*^(motion)^_*j*,*α*_.

Accordingly, the contribution of a particular motional group to a given normal mode *j* is defined by the sum of squares 
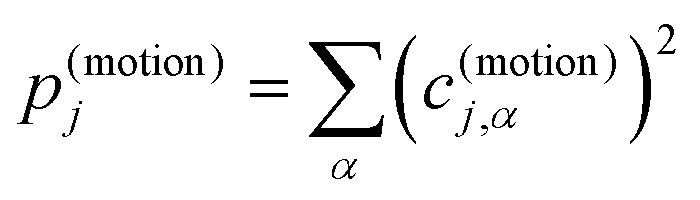
 which is subsequently employed as a weighting factor when computing the partial vibrational DOS; this is analogous to the commonplace partial DOS based on atomic displacement contributions. The vibrational projection analysis reveals that the low-energy DOS indeed features vibrations which have significant inter-fragment character. Upon introducing alkyl bridges between the ligands, the inter-fragment contribution to vibrations in the 0–100 cm^−1^ regime witnesses a significant dampening. At the same time, the contribution of intra-molecular vibrations flattens out towards the low-energy end. Overall, the decrease of the low-energy vibrational DOS can be attributed to increased inter- as well as intra-fragment rigidity of the ligand cage introduced by covalent linkers between the Cp^R^ ligands. This further promotes the ligand system increasingly acting as a single bidentate coordinating unit, as the inter-fragment coupling is accompanied by a splitting of the inter-fragment vibrations into blue-shifted out-of-phase combinations and red-shifted in-phase combinations of the independent Cp^R^ motion. The former vibrations involve significant strain along increasingly rigid DOFs of the ligand cage, while the latter preserve these DOFs. As a result, this raises the energy of intra-fragment and out-of-phase inter-molecular vibrations while lowering the energy of the in-phase inter-fragment vibrations, which leads to a slow increase of the DOS at low energies, which precisely follows the guidelines to suppress two-phonon Raman relaxation.^9,19^

### 3.2 Modelling of magnetic relaxation in the condensed phase

Low-temperature magnetic relaxation dynamics is driven by low-energy (pseudo-)acoustic phonons, which makes a condensed phase model of any real or proposed SMM imperative. However, the quantum mechanical description of an extended environment quickly gets prohibitively expensive with increasing system size. To mimic macroscopic chemical systems, periodic boundary conditions are a common choice. In this way, artificial boundaries containing the molecule(s) of interest are avoided, and instead an infinite array of repeating unit cells is constructed. The present study focusses on the accurate description of the phonons as well as the spin states of the SMM. While the latter demands a QM description, the inter-nuclear potential can be well-approximated by MM.^46^ Here we employ the popular ONIOM^23,47^ QM/MM protocol for structural and vibrational modelling, followed by CASSCF calculations including explicit solvent molecules in the first unit cell and a continuum model to capture the long-range solvent effects; we have recently shown the latter method is capable of achieving experimental accuracy for magnetisation dynamics in molecular crystals.^16^

**Fig. 4:**
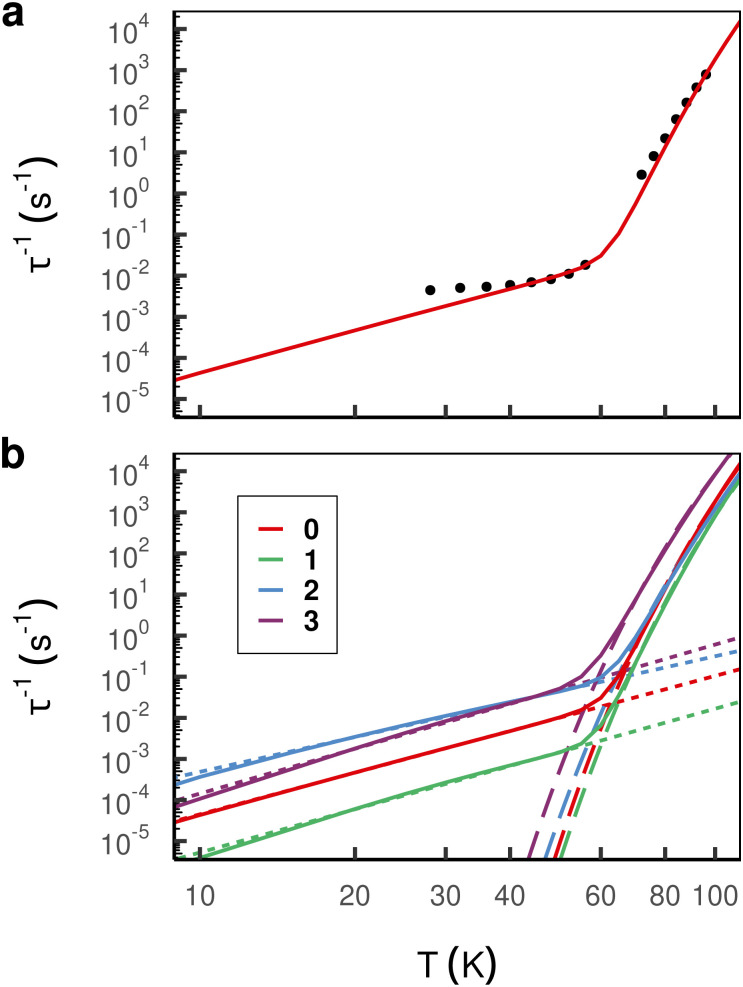
(a) Comparison of simulated total (Orbach + Raman) relaxation rates (red line) and experimental rates of compound 0 (black dots) taken from.^1^ Close agreement is observed above ∼40 K, whereas at lower temperature experimental rates are dominated by QTM which is not included in the simulation. (b) Simulated total relaxation rates (solid line) and the best fit of Orbach (dashed line) and Raman (dotted line) processes with Arrhenius and power law, respectively.

The magnetic relaxation rates are computed based on a system-bath Hamiltonian parameterised by the electronic state energies, the vibrational frequencies and the spin–phonon couplings derived from first principles calculations (see Section 2 for details). Performing such calculations for the non-bridged parent compound 0, we find excellent agreement with experimental data above ∼40 K (Fig. 4(a)),^1^ while below ∼40 K quantum tunneling of magnetisation (QTM) becomes dominant which is not considered in our spin–phonon calculations.^48^ As expected, our calculations for single-phonon processes produce rates with an exponential temperature dependence that dominate magnetic relaxation at high temperature (*i.e.* these rates represent the Orbach process), and our calculations for two-phonon Raman processes become dominant at low temperatures. These excellent results thus validate our computational approach and give us confidence in our predictions of magnetic relaxation rates for the hypothetical bridged compounds.

Subsequently we perform calculations of the magnetic relaxation rates for the bridged compounds 1–3. While compound 1 shows slower magnetic relaxation than compound 0 at all temperatures, increasing the number of bridges beyond one appears to have a detrimental effect on the magnetic memory time, especially at low temperatures (Fig. 4(b)). To understand these results, we first fit our calculated single-phonon relaxation rates using the phenomenological Arrhenius-like expression for Orbach relaxation, 
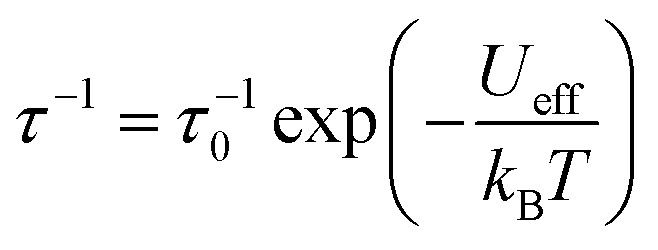
, where *τ*_0_^−1^ and *U*_eff_ are the pre-exponential and effective energy barrier, respectively (Table 1).

**Table 1:** Arrhenius and power-law parameters of the Orbach and Raman processes in the temperature ranges 10 K ≥ *T* ≥60 K as well as 60 K ≥ *T* ≥ 120 K, respectively. Reported uncertainties are the estimate standard errors from the least squares linear fits

SMM	*U*_eff_/cm^−1^	*τ*_0_^−1^/10^9^ S^−1^	*n*	*C*/10^−8^ K^−*n*^ s^−1^
0	1330 ± 10	360 ± 80	3.368 ± 0.007	1.92 ± 0.04
1	1340 ± 10	190 ± 40	3.5 ± 0.06	0.17 ± 0.04
2	1200 ± 20	40 ± 10	2.81 ± 0.04	75 ± 10
3	1150 ± 10	130 ± 30	3.63 ± 0.06	3.4 ± 0.7

**Fig. 5:**
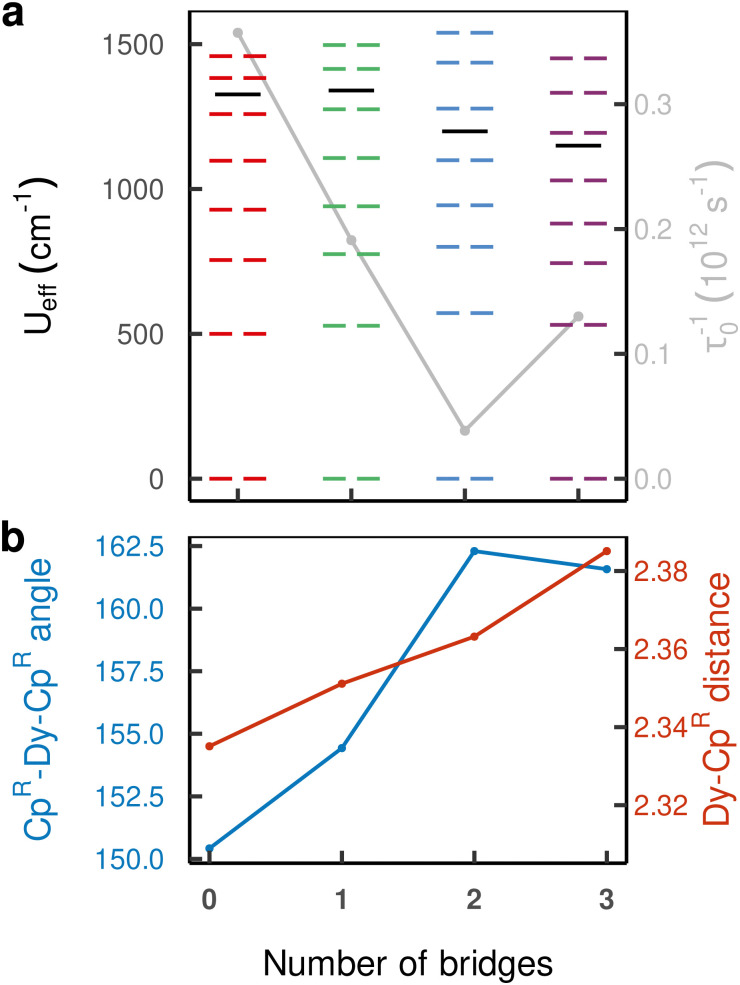
(a) *U*_eff_ (left axis) and *τ*_0_^−1^ (right axis) Arrhenius parameters of the over-barrier Orbach process. The values of the anisotropy barrier are overlayed on the crystal field states constituting the ^6^H_15/2_ multiplet of the respective compound and (b) geometric Cp^R^–Dy–Cp^R^ angle (left axis) and bond length (right axis) parameters.

The effective barrier *U*_eff_ reaches its maximum value already after the introduction of a single bridge and rapidly decreases upon introducing further bridges. This is in contrast with the energy of both the first excited and highest-energy KD which keep increasing until compound 2 (Fig. 5(a)). While the Cp^R^–Dy–Cp^R^ angle increases with additional bridges as expected, its positive effect is counteracted by a simultaneous increase in Dy–Cp^R^ distance, which decreases anisotropy (Fig. 5(b)). Indeed, compound 3 exhibits a decrease in the total ^6^H_15/2_ crystal field splitting resulting from the monotonously rising bond length and stagnating increase in coordination angle. Aside from geometrical effects on the placement of the Cp^R^ rings, the addition of bridges places atoms into the equatorial plane of the Dy(iii) ion, leading to a further reduction in axial magnetic anisotropy, which also explains the progressively increasing under-cutting of the total ^6^H_15/2_ manifold in the effective *U*_eff_ parameter, starting above the third-highest energy KD in 0 and 1, and dropping below the third-highest energy KD for 2 and 3. This is a result of the increasing equatorial component of the crystal field, which leads to increasingly admixed |*m*_J_〉 states throughout the series (see ESI,† Tables S3–S6). The pre-exponential factor *τ*_0_^−1^ decreases traversing the series 0–2 by one order of magnitude, and slightly increases for compound 3 to *ca.* one third of the value found for compound 0.

Performing a similar analysis for the two-phonon rates embodying Raman-like magnetic relaxation, we fit the calculated rates to *τ*^−1^ = *CT*^*n*^, where *C* and *n* are the Raman pre-factor and exponent, respectively (Table 1). While these phenomenological parameters lack the direct physical interpretation of their Orbach counterparts, it has been argued that they are connected to the axiality of the low-lying KDs, the energy gap between the ground and first excited KD, as well as the shape of the vibrational DOS at low energies.^9,19^ Here we find that compound 1 exhibits Raman rates which are consistently around one order of magnitude slower than the non-bridged parent molecule 0 (Fig. 4(b)), which appears to be governed by a significant drop in the pre-factor *C* by *ca.* one order of magnitude, while the exponent *n* is more-or-less the same. Compounds 2 and 3, which have faster magnetic relaxation rates than compound 0 in the Raman regime, have larger *C* and *n* in the case of 3, while 2 has a slightly reduced value for *n* and a 38-fold increase in *C*.

There appears to be no consistent correlation between the number of bridges and the observed Raman rates of compounds 0–3. Indeed, neither the purity of the ground KD (quantified as |〈*Ψ*_KD1_|*m*_J_ = ±15/2〉|^2^ = 99.64% (0), 99.81% (1), 99.91% (2), 99.93% (3)), nor the opening of the energy gap between ground and first excited KD can be identified as correlating factors. However, examination of the purity of the first excited KD (quantified by |〈*Ψ*_KD2_|*m*_J_ = ±13/2〉|^2^ = 99.58% (0), 99.70% (1), 99.47% (2), 99.46% (3)) is maximal for compound 1 and minimal for compounds 2 and 3, and so does correlate with the observed Raman rates.

**Fig. 6:**
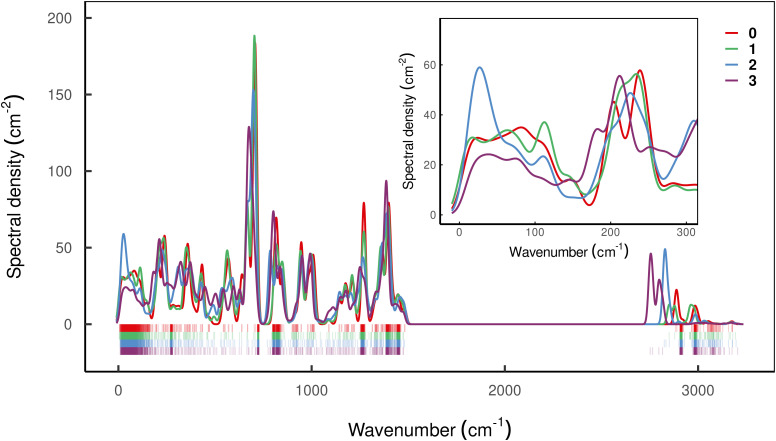
Spectral density characterising vibrations of compound 0–3 in frozen solution. Coloured ticks in the bottom of the plot indicate the position of individual vibrations which are dressed with a Gaussian function of bandwidth 10 cm^−1^ and summed weighted by 
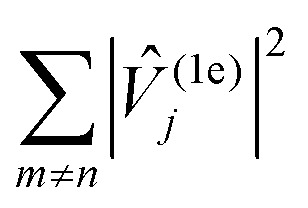
. Highly dense regions in the tick diagram mark the position of solvent bands.

However, having performed first-principles spin–phonon calculation, we can look beyond phenomenological parameters into the atomistic origins for these effects. Compared to the gas-phase vibrational analysis already discussed (Section 3.1), the obvious changes for a frozen solvent model include a large contribution of solvent modes to the DOS as well as rigid-body motions of the SMM which appear as additional DOFs (ESI,† Fig. S3). Additionally, we observe a general blue-shift of all SMM vibrations due to confinement in the solvent cavity (ESI,† Fig. S3 *cf.*Fig. 3), but otherwise the same conclusions about the SMM vibrations can be drawn in the frozen solution phase as for the gas phase. Note, in line with the model of a dilute solution, we only evaluate phonons at the *Γ*-point assuming phonon bands which are sufficiently flat. To analyse the spin–phonon coupling, it is useful to interrogate the atomic motion localised on the SMM which give the strongest coupling to the magnetic states. While vibrational decomposition is one approach to extract SMM vibrational contribution otherwise masked by the prominent phonon bands of the solvent (*e.g.* ESI,† Fig. S3), computing the spectral density (*i.e.* the product of the phonon DOS and the spin–phonon coupling strength per mode, which here we define as the square Frobenius norm of the spin–phonon coupling operator of the respective mode *j* (
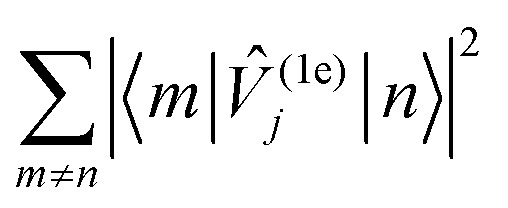
)), gives insight into the presence of strongly coupled modes irrespective of their origin in the vibrational spectrum (Fig. 6). We choose this definition as it resembles the |〈*m*|*V̂*^(1e)^_*j*_|*n*〉|^2^ factor entering the relaxation rate expressions (see eqn (40) and (41) in ref. 15), and we exclude diagonal elements of *V̂*^(1e)^_*j*_ because they do not cause population transfer but merely renormalise state energies. As the two-phonon Raman mechanism solely implicates phonons that couple the states of the ground KD *via* all other states, we can also examine a reduced spectral density that restricts the sum over *m* to the two states of the ground KD (ESI,† Fig. S4). This sheds light onto the spin–phonon coupling patterns of the low-energy vibrations previously shadowed by the (pseudo-)acoustic phonon band dominated by solvent DOFs. While these modes have a substantial contribution to the vibrational DOS owing to their large number of DOFs, they couple weakly and almost exclusively through slight admixture of SMM vibrations; there are no clear bands in the spectral density (Fig. 6) that match primarily solvent modes in the DOS (ESI,† Fig. S3).

Interestingly, all compounds studied here except 2 show rather similar low-energy spectral densities, with compound 3 exhibiting the lowest magnitude spectral density, presumably as a result of the bridging-induced dampening of the DOS in this energy regime. The spectral density of compound 2, however, is quite different, and features a relatively large peak around 30 cm^−1^ which is consistent with its decidedly different Raman relaxation parameters from the other compounds studied here. Furthermore, compound 2 has the largest total spin–phonon coupling strength, which we quantify by computing 
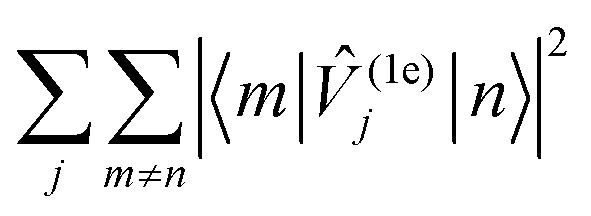
, *i.e.* the integral of the spectral density (see ESI,† Fig. S5a).

**Fig. 7:**
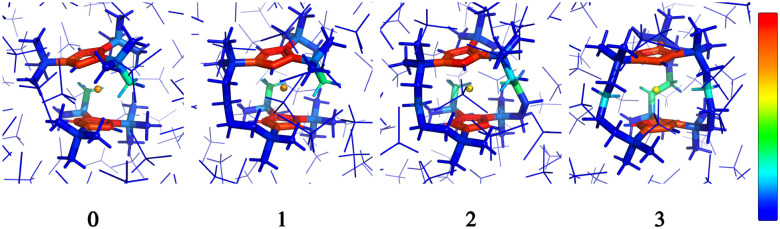
Frozen solution phase geometries of the non-bridged parent dysprosocenium 0 and its ***n***-bridged analogues 1–3. Colouring denote the atomic coupling norms from 0 cm^−1^ Å^−1^ (blue) to 1400 cm^−1^ Å^−1^ (red).

Examination of the atomic couplings 
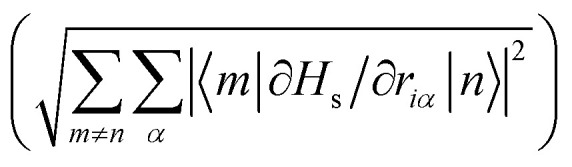
 which is the Euclidean norm of the atomic spin–phonon coupling parameters over all pairs of states *m* ≠ *n* and Cartesian directions (*α* ∈ {*x*,*y*,*z*}) visualised on the equilibrium structure (Fig. 7) shows that spin transfer is mostly induced by atomic motion of the carbon Cp framework and the central Dy(iii) itself. Traversing the series, a drop in the atomic spin–phonon coupling strength at Dy(iii) is observed, 1022, 1023, 901, 817 cm^−1^ Å^−1^ (0–3), which we suspect is due to an increasing distance to the Cp^R^ ligands, as well as an increasingly symmetric coordination environment. Furthermore, the ability of the linker atoms to introduce spin transfer is observed to strongly depend on their respective distance to the Dy(iii) ion; the first linker placed in the equatorial position for compound 1 is facing away from the Dy(iii) ion and exhibits weak coupling. This equatorial coupling increases in magnitude as the increasing Cp^R^–Dy–Cp^R^ angle brings the first linker closer to the Dy(iii) ion throughout the series until coupling magnitudes comparable to the other subsequent aliphatic linker groups are observed. The total atomic spin–phonon coupling, which can be quantified by the square Frobenius norm 
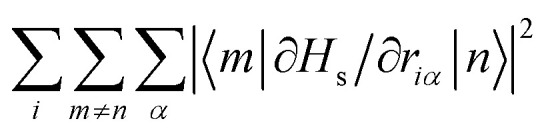
 in analogy to the total spin–phonon coupling strength discussed above, (ESI,† Fig. S5b) reflect these trends showing an initial slight increase in total coupling going from compound 0 to 1 followed by a monotonous decline in 2 and 3.

## 4 Conclusions

The quickly advancing field of SMM design has identified numerous physical parameters that can be optimised to achieve ever longer magnetic memory times. However, it is well known that tweaking a single parameter in isolation often introduces detrimental effect on others. Here we have explored the possibility of bridging the Cp^R^ ligands in cationic Dy(iii) bis-Cp sandwich SMMs. Our first-principles spin–phonon coupling calculations for the existing non-bridged analogue in a frozen DCM matrix show excellent agreement with experimental data. Similar calculations for up to triply-bridged variants demonstrate that the Cp^R^ bridging strategy has potential to suppress both Orbach and Raman rates beyond purely steric design considerations. When one bridge is used, the Orbach mechanism is primarily slowed down due to structural changes that increase the magnetic anisotropy (where the bridge constrains the Cp^R^–Dy–Cp^R^ angle to be more linear), and the Raman mechanism is also slowed down due to both a higher purity first-excited KD as well as enhanced rigidity of the first coordination sphere; note that the latter is unlikely be achieved by solely tuning the Cp-substituents. Among the molecules investigated, the introduction of a single bridge leads to a clear improvement in the magnetic memory time by around one order of magnitude across the temperature range and relaxation processes considered. However, increasing to two or three bridges does not lead to a further increase in the magnetic memory timescale, and instead results in a significant speed-up of magnetic relaxation. While the bridging design principle is successful in progressively reshaping the low-energy DOS, blue-shifting inter-fragment SMM vibrations throughout the series, introducing alkyl linkers in the equatorial plane of the SMMs disrupts the axial magnetic anisotropy that is vital for slow magnetic relaxation. Furthermore, even though the Cp^R^–Dy–Cp^R^ angle increases throughout the series, the Dy–Cp^R^ bond length increases and the effective Orbach barrier is reduced as the purity of the higher-lying KDs decreases. Furthermore, while bridging provides a handle on vibrational frequencies, the strength of coupling of individual modes, *i.e.* the destructive and constructive linear combination of strong atomic couplings governed by the per-mode atomic displacement magnitudes and phases, can not fully be controlled using this approach. Of course our work here proposes only one type of linker for one type of Cp^R^ ligand, and refinement of the bridging strategy could employ different linkers, varying carbon chain length, saturation, aromaticity, and so on. Shorter bridges and double bonds might further enhance ligand rigidity, while the latter might additionally avoid the presence of strongly coupled atoms in the equatorial plane. We have laid the path for such calculations in this work.

From a methodological standpoint, the condensed frozen-solution-phase environment model proposed and successfully employed in this work is an important step towards a generic modelling approach for *in silico* conceived SMMs, and in general for the study of phononic interactions for molecules without known crystal structures, and hence we predict that such methodologies will be crucial for the manual as well as automatic high-throughput exploration of chemical space in fields beyond molecular magnetism.

## Author contributions

JKS and NFC conceived the project. MKR and JKS performed *ab initio* calculations and data analysis. JKS and NFC jointly drafted the manuscript.

## Data availability

All research data is available at DOI: 10.48420/25356166, as stated in the paper.

## Conflicts of interest

There are no conflicts to declare.

## Supplementary Material

CP-026-D4CP01716A-s001
