# Supplementary material for: Intramolecular bridging strategies to suppress two-phonon Raman spin relaxation in dysprosocenium single-molecule magnets
Source: Phys Chem Chem Phys. 2024 Jun 10;26(25):17539–48. doi: 10.1039/d4cp01716a (PMC11202312; doi:10.1039/d4cp01716a)
Supplement: CP-026-D4CP01716A-s001 [file CP-026-D4CP01716A-s001.pdf]

Electronic Supplementary Material (ESI) for Physical Chemistry Chemical Physics.
This journal is © the Owner Societies 2024

Supplementary Information

Intramolecular bridging strategies to suppress two-phonon Raman spin relaxation in dysprosocenium single-molecule magnets

Jakob K. Staab[1], Md. Kholilur Rahman[1] and Nicholas F. Chilton*[1,2]

[1]Department of Chemistry, The University of Manchester, Manchester M13 9PL, U.K.
[2]Research School of Chemistry, The Australian National University, Canberra 2601, ACT, Australia

June 5th, 2024

Contents

S1 Classical force field interactions 2

S2 Synthetic strategy 4

S3 Magnetic relaxation 5

S4 Characterisation of the crystal field states 6

S5 Condensed phase vibrational DOS and coupling strength 9

*email: nicholas.chilton@manchester.ac.uk

S1 Classical force field interactions

The OPLS-AA forcefield is employed to describe all bonded and nonbonded interactions. Since quantum mechanics (QM) and molecular mechanics (MM) region solely interact through nonbonded atomic van der Waals (vdW) and coulomb interactions, only the inter-molecular solvent interactions include harmonic covalent bond and angle stretching contributions involving force constants $k_{r,\theta}$ and equilibrium bond lengths and angles r_0 and θ_0, see Table S1.

$$E_{\text{bonded}} = \sum_{\text{bonds}} k_r (r - r_0)^2 + \sum_{\text{angles}} k_\theta (\theta - \theta_0)^2 \tag{1}$$

All nonbonded interactions expressed in Equation 2 involve atomic charges $\{q_i\}$ to describe Coulomb interactions, and $\{\epsilon_{ij}\}$ and $\{\sigma_{ij}\}$ parameters to describe vdW interactions using the geometric mean combination rule for hetero-atomic interactions between atom types i and j ($\epsilon_{ij} = \sqrt{\epsilon_{ii}\epsilon_{jj}}$ and $\sigma_{ij} = \sqrt{\sigma_{ii}\sigma_{jj}}$). All nonbonded parameters are compiled in Table S2.

$$E_{\text{nonbonded}} = \sum_{\langle i,j \rangle} \frac{q_i q_j}{r_{ij}} + 4\epsilon_{ij} \left(\frac{\sigma_{ij}^{12}}{r_{ij}^{12}} - \frac{\sigma_{ij}^{6}}{r_{ij}^{6}} \right) \tag{2}$$

where r_{ij} are the interatomic distances and the notation $\langle i, j \rangle$ indicates pairs of atoms being restricted to different molecules.

Table S1: DCM bonded force field parameters.

atoms	k_r/kcal mol^{-1} Å^{-2}	r_0/Å
C−H	340.0	1.09
C−Cl	245.0	1.781
atoms	k_θ/kcal mol^{-1} rad^{-2}	θ_0/°
H−C−H	33.0	107.8
Cl−C−Cl	78.0	111.7
H−C−Cl	51.0	107.6

Table S2: Nonbonded force field parameters.

atom	charge	σ_{ii}/Å	ϵ_{ii}/kcal mol^{-1}
DCM			
C	−0.006	3.905	0.118
H	0.103	3.8	0.08
Cl	−0.1	3.4	0.3
$[Dy(Cp^{ttt})_2]^+$			
Dy	–[a]	3.5	0.066
Cp−C	–[a]	3.8	0.05
alkly−C	–[a]	3.905	0.118
Cp−H	–[a]	3.47	0.26
alkly−H	–[a]	3.8	0.08

[a] Atomic charges fitted to density-functional theory (DFT) density. See data repository.

S2 Synthetic strategy

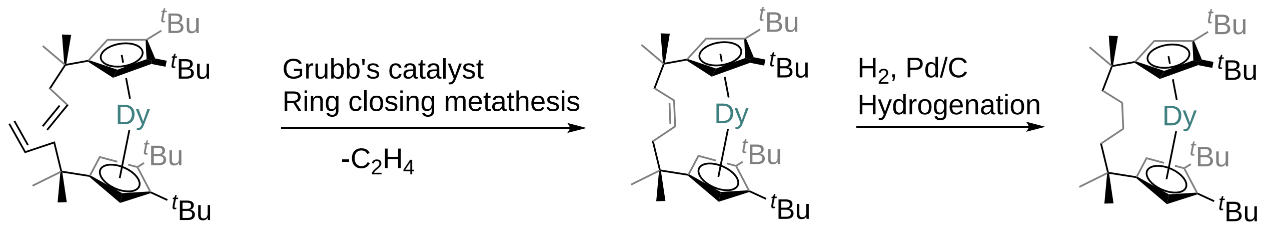

1

Figure S1: Possible synthetic route of alkyl-bridged $[Dy(Cp^R)_2]^+$ molecules via a ring-closing metathesis reaction of two alkene-tethered Cp^R ligands and a subsequent hydrogenation.

S3 Magnetic relaxation

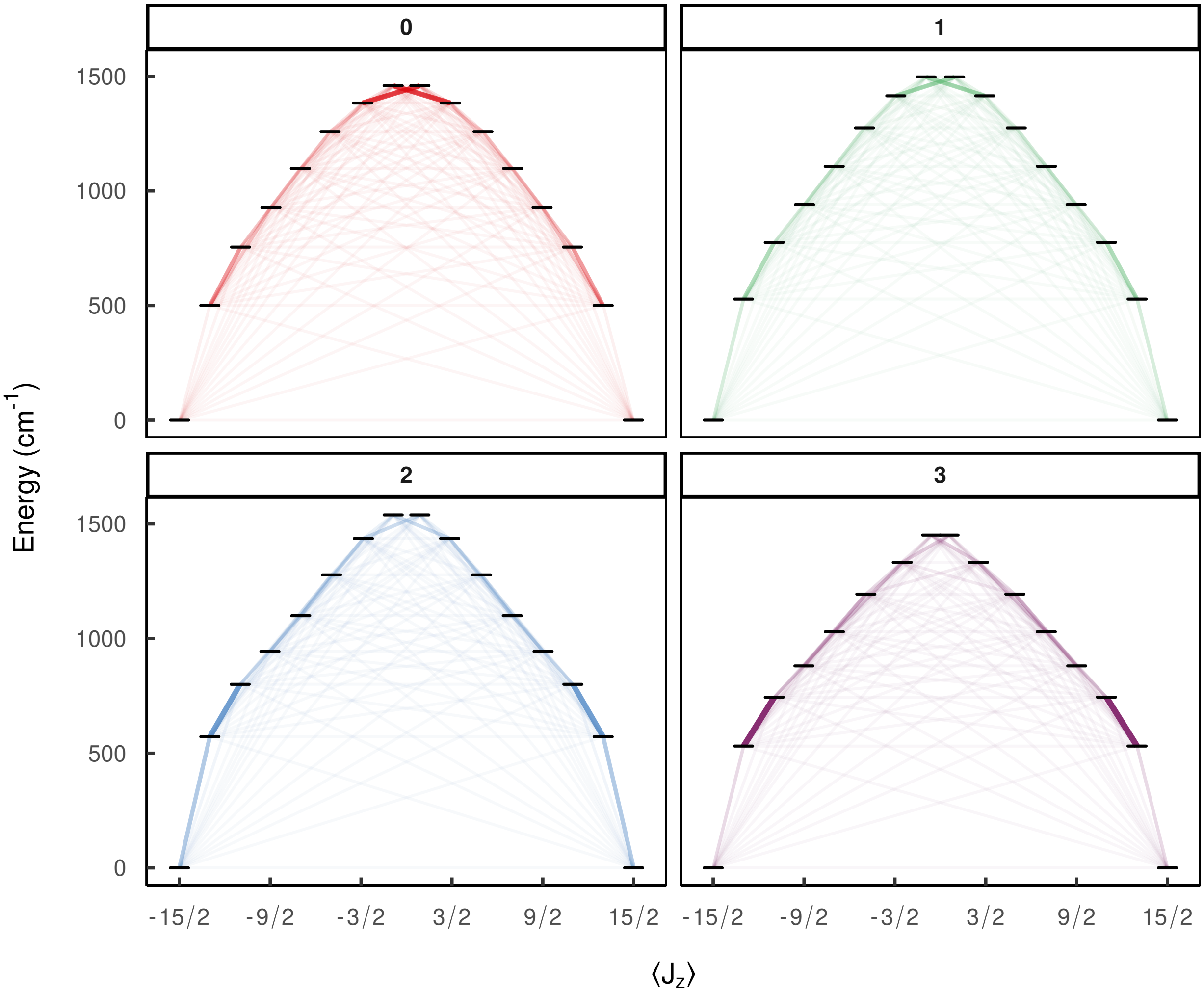

Figure S2: Barrier diagrams depicting Orbach transition rates between the magnetic sub-levels at 100 K.

S4 Characterisation of the crystal field states

Table S3: Kramers doublets of $[\mathrm{Dy(Cp^{ttt})_2}]^+$ (**0**) under application of a magnetic field $B_z = 0.2\,\mathrm{mT}$ as obtained from the crystal field parameterisation of a (9,7)-CASSCF calculation (see main text Section 2 for details).

E/cm^{-1}	$\langle J_z \rangle$	g_1	g_2	g_3	State composition
0.00	±7.49	0.00	0.00	19.99	99.64% $\vert\pm15/2\rangle$, 0.35% $\vert\pm11/2\rangle$
500.22	±6.49	0.00	0.00	17.35	99.58% $\vert\pm13/2\rangle$, 0.36% $\vert\pm11/2\rangle$
754.90	±5.48	0.00	0.00	14.64	97.25% $\vert\pm11/2\rangle$, 1.05% $\vert\pm7/2\rangle$, 0.95% $\vert\pm9/2\rangle$, 0.36% $\vert\pm13/2\rangle$, 0.32% $\vert\pm15/2\rangle$
928.84	±4.47	0.01	0.02	11.99	97.24% $\vert\pm9/2\rangle$, 1.61% $\vert\pm5/2\rangle$, 0.97% $\vert\pm11/2\rangle$
1097.68	±3.50	0.12	0.15	9.38	97.94% $\vert\pm7/2\rangle$, 1.01% $\vert\pm11/2\rangle$, 0.78% $\vert\pm3/2\rangle$, 0.15% $\vert\pm9/2\rangle$
1258.69	±2.52	0.67	1.04	6.74	97.98% $\vert\pm5/2\rangle$, 1.60% $\vert\pm9/2\rangle$, 0.16% $\vert\mp3/2\rangle$
1383.19	±1.45	2.49	3.89	4.89	95.74% $\vert\pm3/2\rangle$, 3.03% $\vert\mp1/2\rangle$, 0.81% $\vert\pm7/2\rangle$, 0.20% $\vert\mp5/2\rangle$, 0.11% $\vert\pm1/2\rangle$
1458.80	±0.44	1.17	6.66	13.98	96.60% $\vert\pm1/2\rangle$, 3.01% $\vert\mp3/2\rangle$, 0.15% $\vert\pm3/2\rangle$

Table S4: Kramers doublets of $[Dy(Cp^{ttb})_2]^+$ (**1**) under application of a magnetic field $B_z = 0.2\,mT$ as obtained from the crystal field parameterisation of a (9,7)-CASSCF calculation (see main text Section 2 for details).

E/cm^{-1}	$\langle J_z \rangle$	g_1	g_2	g_3	State composition
0.00	±7.50	0.00	0.00	20.00	99.81% $\lvert\pm15/2\rangle$, 0.18% $\lvert\pm11/2\rangle$
528.09	±6.50	0.00	0.00	17.35	99.70% $\lvert\pm13/2\rangle$, 0.26% $\lvert\pm11/2\rangle$
775.29	±5.48	0.00	0.00	14.65	98.07% $\lvert\pm11/2\rangle$, 0.74% $\lvert\pm7/2\rangle$, 0.72% $\lvert\pm9/2\rangle$, 0.26% $\lvert\pm13/2\rangle$, 0.17% $\lvert\pm15/2\rangle$
940.52	±4.48	0.01	0.01	11.99	98.04% $\lvert\pm9/2\rangle$, 1.06% $\lvert\pm5/2\rangle$, 0.75% $\lvert\pm11/2\rangle$, 0.11% $\lvert\pm7/2\rangle$
1106.96	±3.50	0.08	0.10	9.37	98.49% $\lvert\pm7/2\rangle$, 0.71% $\lvert\pm11/2\rangle$, 0.57% $\lvert\pm3/2\rangle$, 0.15% $\lvert\pm9/2\rangle$
1275.19	±2.51	0.97	1.20	6.69	98.34% $\lvert\pm5/2\rangle$, 1.05% $\lvert\pm9/2\rangle$, 0.26% $\lvert\mp3/2\rangle$, 0.20% $\lvert\pm1/2\rangle$
1414.54	±1.47	1.48	3.75	3.94	97.45% $\lvert\pm3/2\rangle$, 1.48% $\lvert\mp1/2\rangle$, 0.59% $\lvert\pm7/2\rangle$, 0.33% $\lvert\mp5/2\rangle$
1496.95	±0.47	1.26	7.90	13.14	98.14% $\lvert\pm1/2\rangle$, 1.54% $\lvert\mp3/2\rangle$, 0.15% $\lvert\pm5/2\rangle$

Table S5: Kramers doublets of $[Dy(Cp^{tbb})_2]^+$ (**2**) under application of a magnetic field $B_z = 0.2\,\mathrm{mT}$ as obtained from the crystal field parameterisation of a (9,7)-CASSCF calculation (see main text Section 2 for details).

E/cm^{-1}	$\langle J_z \rangle$	g_1	g_2	g_3	State composition
0.00	±7.50	0.00	0.00	20.01	99.91% $\lvert\pm15/2\rangle$
571.96	±6.49	0.00	0.00	17.35	99.47% $\lvert\pm13/2\rangle$, 0.43% $\lvert\pm11/2\rangle$
800.53	±5.49	0.01	0.02	14.68	98.25% $\lvert\pm11/2\rangle$, 1.21% $\lvert\pm9/2\rangle$, 0.42% $\lvert\pm13/2\rangle$
943.86	±4.51	0.03	0.06	12.07	98.37% $\lvert\pm9/2\rangle$, 1.18% $\lvert\pm11/2\rangle$, 0.24% $\lvert\pm7/2\rangle$
1099.63	±3.49	0.62	0.69	9.37	99.48% $\lvert\pm7/2\rangle$, 0.23% $\lvert\pm9/2\rangle$, 0.11% $\lvert\mp5/2\rangle$
1277.66	±2.48	0.26	1.61	6.64	99.13% $\lvert\pm5/2\rangle$, 0.31% $\lvert\pm1/2\rangle$, 0.20% $\lvert\mp3/2\rangle$, 0.13% $\lvert\pm3/2\rangle$, 0.10% $\lvert\mp7/2\rangle$
1436.38	±1.42	2.93	3.80	4.81	95.84% $\lvert\pm3/2\rangle$, 3.38% $\lvert\mp1/2\rangle$, 0.29% $\lvert\pm1/2\rangle$, 0.23% $\lvert\mp5/2\rangle$, 0.11% $\lvert\pm5/2\rangle$
1539.46	±0.44	1.16	6.60	14.34	95.72% $\lvert\pm1/2\rangle$, 3.34% $\lvert\mp3/2\rangle$, 0.36% $\lvert\pm3/2\rangle$, 0.29% $\lvert\pm5/2\rangle$, 0.16% $\lvert\mp1/2\rangle$

Table S6: Kramers doublets of $[Dy(Cp^{bbb})_2]^+$ (**3**) under application of a magnetic field $B_z = 0.2\,\mathrm{mT}$ as obtained from the crystal field parameterisation of a (9,7)-CASSCF calculation (see main text Section 2 for details).

E/cm^{-1}	$\langle J_z \rangle$	g_1	g_2	g_3	State composition
0.00	±7.50	0.00	0.00	20.01	99.93% $\lvert\pm 15/2\rangle$
531.17	±6.49	0.00	0.00	17.35	99.46% $\lvert\pm 13/2\rangle$, 0.50% $\lvert\pm 11/2\rangle$
744.18	±5.48	0.00	0.01	14.66	97.63% $\lvert\pm 11/2\rangle$, 1.47% $\lvert\pm 9/2\rangle$, 0.46% $\lvert\pm 13/2\rangle$, 0.36% $\lvert\pm 7/2\rangle$
880.81	±4.50	0.05	0.06	12.03	97.82% $\lvert\pm 9/2\rangle$, 1.33% $\lvert\pm 11/2\rangle$, 0.58% $\lvert\pm 7/2\rangle$, 0.19% $\lvert\pm 5/2\rangle$
1029.72	±3.50	0.91	1.08	9.41	98.74% $\lvert\pm 7/2\rangle$, 0.47% $\lvert\pm 11/2\rangle$, 0.46% $\lvert\pm 9/2\rangle$, 0.26% $\lvert\mp 5/2\rangle$
1193.80	±2.46	0.28	1.62	6.62	98.14% $\lvert\pm 5/2\rangle$, 1.19% $\lvert\pm 1/2\rangle$, 0.26% $\lvert\mp 7/2\rangle$, 0.19% $\lvert\pm 9/2\rangle$, 0.12% $\lvert\pm 3/2\rangle$
1332.25	±1.26	3.35	6.49	6.89	88.01% $\lvert\pm 3/2\rangle$, 11.26% $\lvert\mp 1/2\rangle$, 0.39% $\lvert\mp 5/2\rangle$, 0.15% $\lvert\pm 5/2\rangle$, 0.11% $\lvert\pm 1/2\rangle$
1451.15	±0.29	0.76	3.27	16.74	87.14% $\lvert\pm 1/2\rangle$, 11.44% $\lvert\mp 3/2\rangle$, 0.84% $\lvert\pm 5/2\rangle$, 0.28% $\lvert\pm 3/2\rangle$, 0.28% $\lvert\mp 1/2\rangle$

S5 Condensed phase vibrational DOS and coupling strength

In the following, the apparent discrepancy between trends observed in the total atomic and spin-phonon coupling strengths shown in Figure S5 is explained. Recalling the definition of the

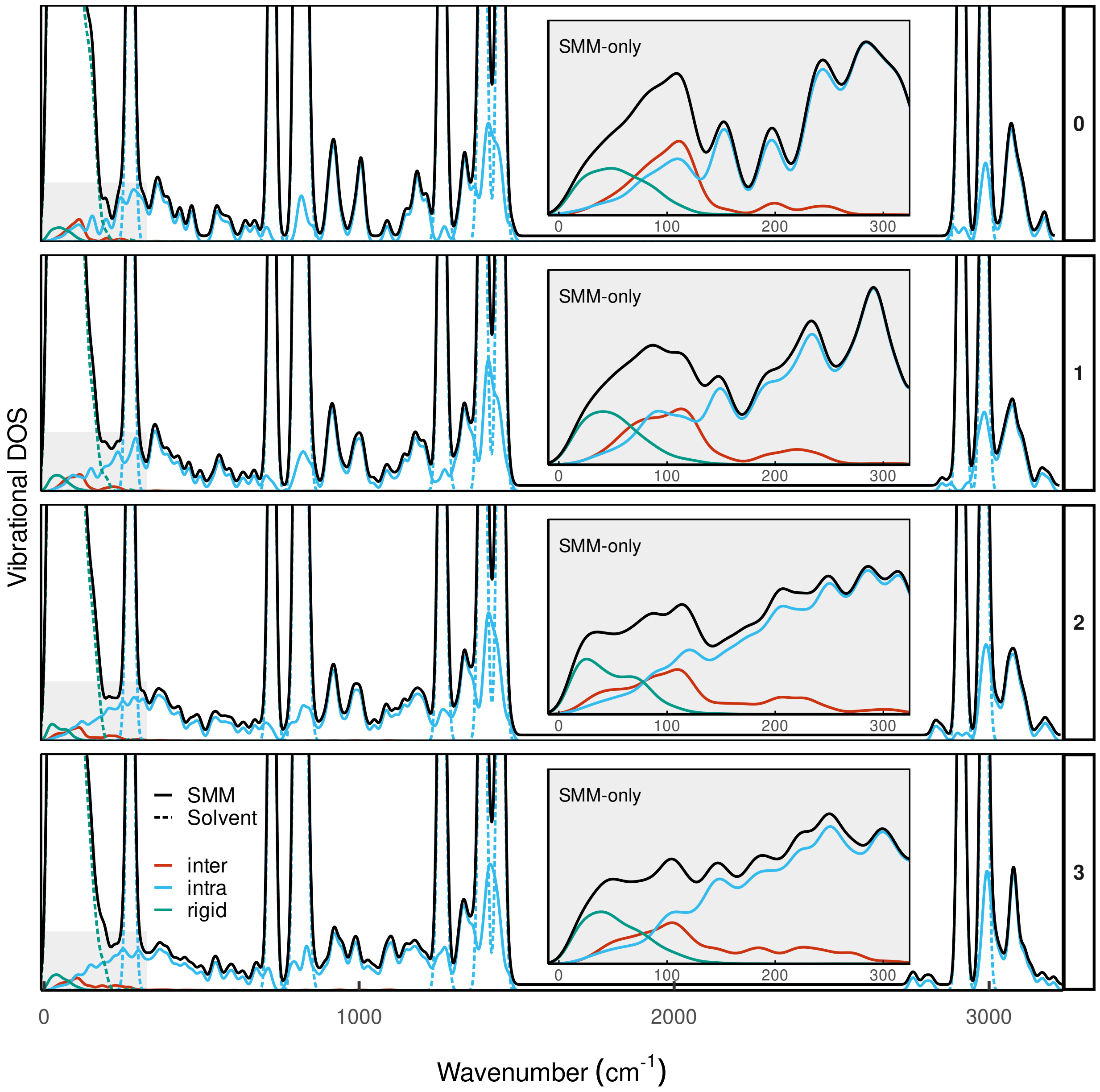

Figure S3: Decomposition of the condensed phase vibrational density of states (DOS) computed with a bandwidth of 10 cm^{-1} into rigid body, inter- and intra-fragment contributions of the single-molecule magnet (SMM) (coloured solid lines) as well as rigid body and intra-molecular vibrations of the solvent molecules (coloured dashed lines) enveloped by the total DOS (black solid line). The total DOS is slightly offset for visualisation purposes. The inset shows the contribution of SMM motion to the low-energy DOS below 300 cm^{-1}

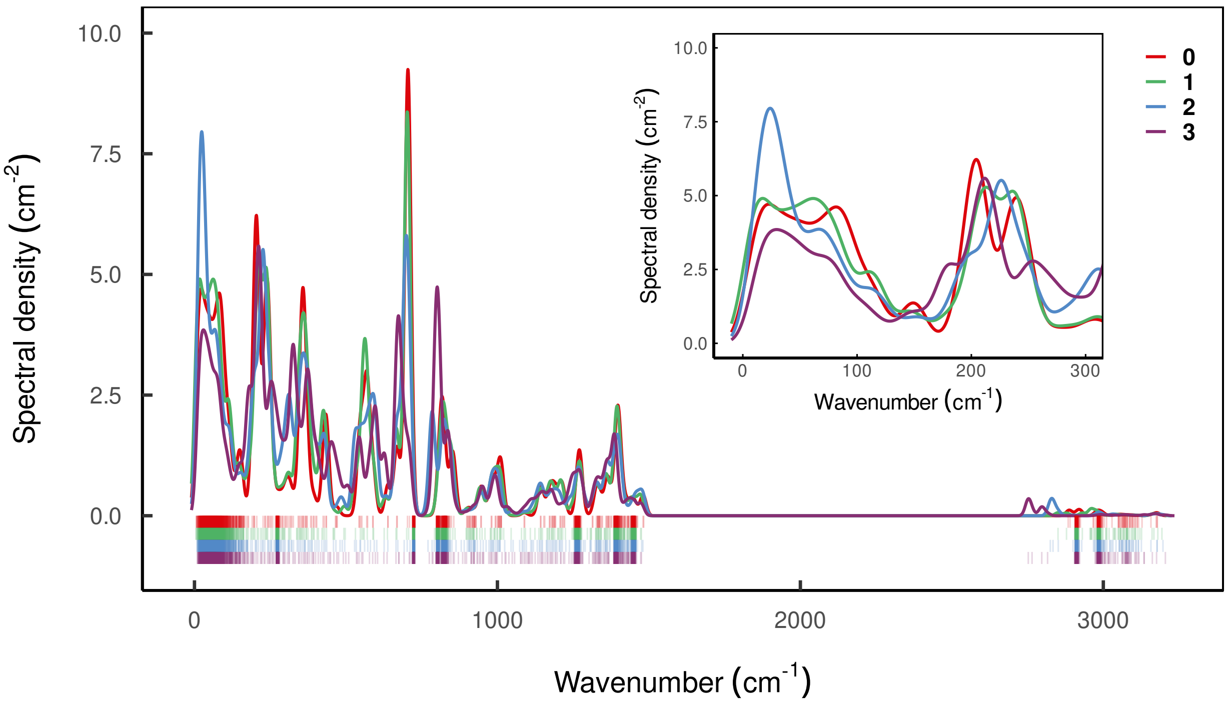

Figure S4: Ground Kramers doublet (KD) spectral density characterising vibrations of compound **0-3** in frozen solution. Coloured ticks in the bottom of the plot indicate the position of individual vibrations which are dressed with a Gaussian function of bandwidth $10\,\mathrm{cm}^{-1}$ and summed weighted by $\sum_{m\in\mathrm{KD}_1}\sum_{n\neq m}|\langle m|\hat{V}_j^{(1e)}|n\rangle|^2$. Highly dense regions in the tick diagram mark the position of solvent bands.

transformation of the spin-phonon coupling matrix elements from atomic to mass-frequency scaled normal mode coordinates defined in ref [1]

$$\langle m|\hat{V}_j^{(1\mathrm{e})}|n\rangle = \left(\frac{\partial H}{\partial X_j}\right)_{mn} = \sqrt{\frac{\hbar}{2\omega_j}} \sum_{i\alpha} \frac{(\boldsymbol{DL})_{i\alpha,j}}{\sqrt{M_i}} \left\langle m \left| \frac{\partial H_{\mathrm{s}}}{\partial r_{i\alpha}} \right| n \right\rangle , \tag{3}$$

this discrepancy can be linked to the $\sqrt{\omega_j}^{-1}$ and $\sqrt{M_i}^{-1}$ factors appearing in Equation 3, where ω_j and M_i are radial frequency of mode j and atomic mass of atom i, respectively, and is a result of transformation 3 being non-orthogonal and hence lacking the norm-preserving property; while the matrix $\boldsymbol{DL}$ (the orthogonal transformation $\boldsymbol{L}$ which diagonalises the mass-weighted Hessian within the non-translating coordinate frame $\boldsymbol{D}$) exhibits this property, the mode and atom specific factors named above render the overall transformation non-orthogonal. The observation that transforming from atomic to normal mode coordinates, the basis relevant for spin-phonon coupling applications, skews the maximum of the total coupling strength magnitude as a function of the number of bridges n towards higher n (Figure S5), further implies that with increasing n, magnitude of the coupling strength is increasingly shifted onto (i) lower frequency vibrations which (ii) naturally involve the motion of heavier atoms, yielding overall lower total spin-phonon coupling strengths at high n.

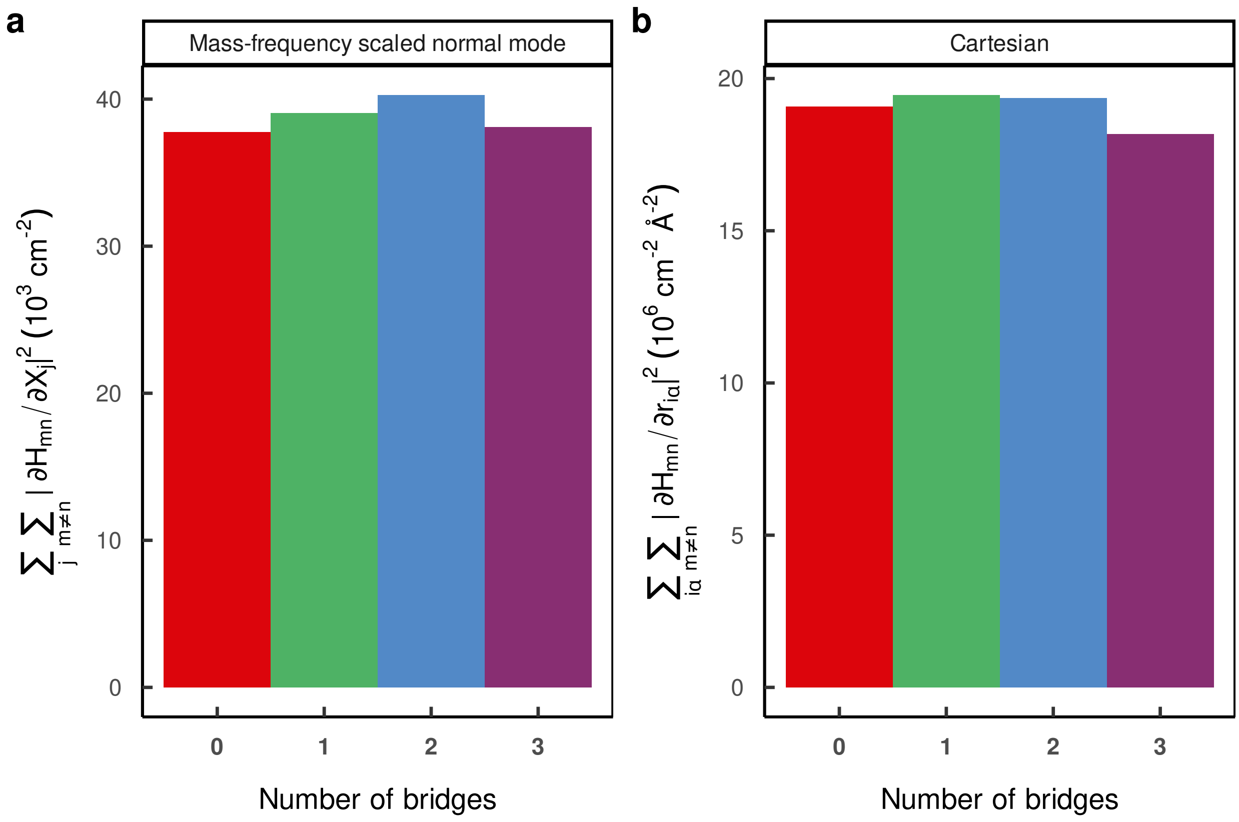

Figure S5: Total coupling strength metric in the normal mode (**a**) and atomic (**b**) basis. (**a**) corresponds to the integral of the spectral density shown in Figure 6 in the main text.

References

(1) Kragskow, J. G. C.; Mattioni, A.; Staab, J. K.; Reta, D.; Skelton, J. M.; Chilton, N. F. Spin–phonon coupling and magnetic relaxation in single-molecule magnets. *Chem. Soc. Rev.* **2023**, *52*, 4567–4585, DOI: 10.1039/d2cs00705c.
